# Renal Replacement Therapy in Children in the Developing World: Challenges and Outcome in a Tertiary Hospital in Southeast Nigeria

**DOI:** 10.1155/2014/903151

**Published:** 2014-11-11

**Authors:** Odutola Israel Odetunde, Henrietta Uche Okafor, Samuel Nkachukwu Uwaezuoke, Bertilla Uzoma Ezeonwu, Oluchi Mildred Ukoha

**Affiliations:** ^1^Pediatric Nephrology Unit, Department of Pediatrics, University of Nigeria Teaching Hospital, PMB 01129, Enugu 400001, Nigeria; ^2^Department of Pediatrics, Federal Medical Centre, Asaba 320212, Delta State, Nigeria; ^3^Department of Pediatrics, Enugu State University Teaching Hospital, Enugu 400261, Enugu State, Nigeria

## Abstract

A 5-year observational, retrospective study was conducted to evaluate the indications, the availability, the accessibility, the sustainability, and the outcome of children managed for acute kidney injury (AKI) and end stage kidney disease (ESKD) who required renal replacement therapy RRT in Enugu, southeast Nigeria. A total of 64 patients aged 5 months to 16 years required RRT, of which only 25 underwent RRT, giving an RRT accessibility rate of 39.1%. Eleven (44%) patients required chronic dialysis program/ renal transplant, of which only 1 (9.1%) accessed and sustained chronic hemodialysis, giving a dialysis acceptance rate of 9.1%. Fifty (78%) of the patients belonged to the low socioeconomic class. Thirty-three (51.5%) could not access RRT because of financial constraints and discharge against medical advice (DAMA); 6 (9.4%) died on admission while sourcing for funds to access the therapy; 5 (7.8%) died while on RRT; 9 (14.1%) improved and were discharged for follow-up; 1 (1.6%) improved and was discharged to be on chronic dialysis program while awaiting renal transplantation outside the country/clinic follow-up, while the remaining 10 (15.6%) were unable to sustain chronic dialysis program or access renal transplantation and were lost to follow-up. We conclude that RRT remains unaffordable within the subregion.

## 1. Introduction

Renal replacement therapy (RRT) is the process of supporting renal function through the application of intermittent or continuous extracorporeal (hemodialysis) or paracorporeal (peritoneal dialysis) methods or ultimately by renal transplantation [1].

The primary indication for RRT is acute or chronic renal failure. The Kidney Dialysis Outcomes Initiative's (K/DOQI) clinical practice guidelines for chronic kidney disease have defined end stage kidney disease (ESKD) as Stage 5 CKD with glomerular filtration rate (GFR) of <15 mL/min/1.73 m^2^ or the use of regular dialysis [2]. Although the Acute Dialysis Quality Initiative (ADQI) group published a consensus definition of acute renal failure (AKI) for adults [3], with a graded severity of Risk, Injury, Failure Loss, and ESKD (RIFLE), this was modified for use in pediatric patients [4]. Other indications for RRT in the hospitalized patient include severe acidosis, hyperkalemia or other electrolyte abnormalities, toxin/poisonings, and fluid overload of greater than ten percent in the critically ill patient [1].

The selection of RRT modality, either peritoneal or hemo-based, depends on patient's characteristics such as age, severity of illness, comorbid illness, and indications for RRT like ion removal, middle molecule clearance, and fluid removal; location which could be inpatient, intensive care unit (ICU), or outpatient; and available resources like financial resources, equipment, and trained personnel [1]. In most developing countries and regions of the world, peritoneal dialysis remains the predominant method of RRT especially in children due to its relative ease and lower cost when compared to hemodialysis [5–7]. Renal transplantation is the ultimate modality of RRT for end stage renal disease, as it replaces native renal function completely and gives near normal quality of life, but nonavailability of organ donors limits its widespread use.

Access to RRT in developed countries keeps over 2 million people alive worldwide [8], while many of the developing countries have no readily accessible and affordable RRT program; so patients who require it end up with a fatal outcome or must travel outside the country for treatment [9–12]. RRT in children remains inaccessible in developing countries such as Nigeria. With the dearth of data from the country, it may be difficult to formulate policies to improve the acceptance rate of RRT in the subregion. In this study, we evaluated the indications, the availability, the accessibility, the sustainability, and the outcome of RRT in children in Enugu, southeast Nigeria.

## 2. Methods

### 2.1. Study Site

This study was carried out at the Pediatric Nephrology Unit of the University of Nigeria Teaching Hospital (UNTH), Enugu. The hospital is among the first generation tertiary hospital facilities in Nigeria with multidisciplinary inpatient and outpatient services. The Pediatric Nephrology Unit of UNTH renders services to patients from the catchment area, predominantly from the southeastern region of Nigeria that has an estimated combined population of 16 million (16,395,552) in which children constitute about 40% of the population [13]. Ethical approval was sought from Health Research and Ethics Committee of UNTH, Enugu, before commencing the study.

### 2.2. Study Methods

The clinical data of children with AKI and ESKD admitted to the pediatric wards who required RRT, from November 2008 to October 2013 (5 years), were reviewed retrospectively. The data obtained included the age, gender, family socioeconomic class (SECS), indications for dialysis, treatment option offered to the patient, the accessibility of the treatment option offered, and the management outcome. The family socioeconomic class (SECS) was determined using the method recommended by Oyedeji to assign socioeconomic class to pediatric patients on hospital admission [14]. The RRT modality choices depended on the age and weight of the patient at presentation.

### 2.3. Definitions

The following definitions are used for the purpose of this study.

#### 2.3.1. RRT Requirement Rate

This was calculated as the number of patients that required RRT during the study period divided by the total number of children aged 2 months to 16 years admitted to the pediatrics wards of the hospital for renal disease during the study period per 100-children population.

#### 2.3.2. RRT Access Rate

RRT access rate was calculated as the total number of patients on admission that accessed RRT during the study period divided by the number of patients that required it per 100 children.

#### 2.3.3. Dialysis Acceptance Rate

This is the rate of chronic dialysis acceptability and is calculated as the number of patients with ESKD who received dialysis divided by the number of children admitted to the pediatric wards of the hospital during the study with the diagnosis of ESKD per 100 children.

### 2.4. Statistical Analysis

Data collected were entered into Microsoft Excel worksheet and were subjected to analysis using the Statistical Package for Social Scientists (SPSS) version 17.0 and results were presented as tables and charts. Descriptive statistics were generated from the outcome variables. Data was subjected to parametric and nonparametric tests.

## 3. Results

**Figure 1:**
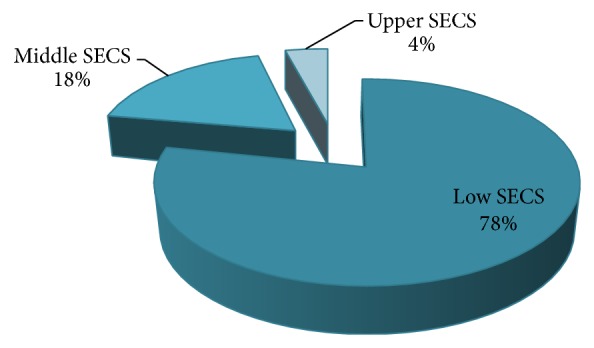
Family socioeconomic class (SECS) distribution of patients.

**Figure 2:**
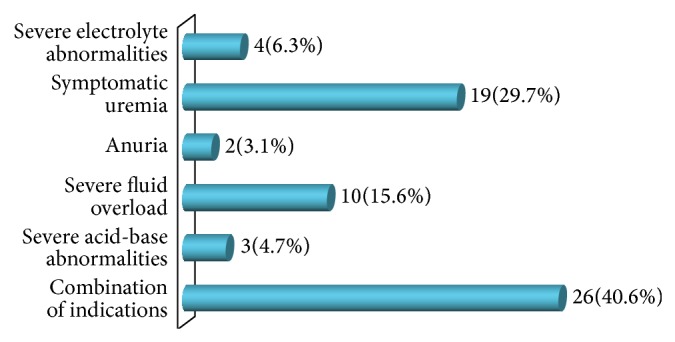
Indication for RRT in patients who required it.

A total of 3,520 patients were admitted to the pediatric wards of the hospital in the period under review (from November 1, 2008, to October 30, 2013) in which 194 patients had different forms of renal disease. Of the 194 patients with renal disease, 64 (33%) patients required RRT; they were aged 5 months to 16 years (mean age of 5.3 years). There were 35 males and 29 females, giving a male to female ratio of 1.2 : 1 (Table 1). Patients were predominantly (78%) of the lower social class (Figure 1). AKI was the commonest diagnosis in patients that required RRT and accounted for 41 (64.1%) of the patients while ESRD accounted for 23 (35.9%) as shown in Table 2. The etiology of the AKI in these patients is as shown in Table 3. Of these 41 patients that had AKI, 3 (4.7%) had acute chronic kidney disease, while 5 (7.8%) had AKI 2^0^ toxic nephropathy (Table 3). Combinations of two or more factors such as severe fluid overload, anuria, severe acid-base abnormalities, symptomatic uremia, and severe electrolyte abnormalities were the major indications for RRT in 26 (40.6%) of the patients as illustrated in Figure 2. Twenty-five (39.1%) of the 64 patients that required RRT accessed the therapy and 39 (60.9%) could not do so because of financial constraints (Table 2).

Patients less than 8 years weighing less than 26 kilograms were placed on acute peritoneal dialysis (APD) while patients aged more than 8 years weighing more than 26 kilograms were placed on acute hemodialysis (AHD). The center used the age and the weight of the patients to determine the modality of dialysis because of availability of materials and consumables needed for the treatment in the region.

**Figure 3:**
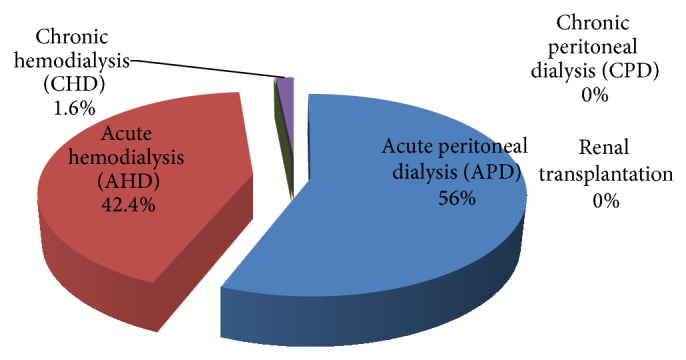
Modality of RRT accessed by patients.

Fourteen (56.0%) of those who accessed RRT received acute peritoneal dialysis (APD) and 11 (44.0%) received acute hemodialysis (AHD) as shown in Figure 3. A total of 23 patients (35.9%) were diagnosed with ESKD; 7 (10.9%) of these patients were in the age range of 8–16 years (mean 12.3 ± 2.9 years) and received AHD, while the remaining 16 (25%) did not receive any form of RRT. A total of 41 (64.1%) presented with AKI; 14 (21.9%) of these patients were within the age of 5 months to 7 years (mean 2.7 ± 2.3 years) and received APD, while 4 (6.3%) patients with AKI were within the age range of 8–16 years (mean 13.8 ± 3.9 years) and received AHD. Four (6.3%) of the patients were admitted with AKI, out of whom 2 (3.1%) presented with toxic nephropathy (1 received APD, while 1 received AHD) and subsequently experienced rapid deterioration of renal function leading to ESKD. Two (3.1%) who presented with AKI on chronic kidney disease 2^0^ CAKUT showed progression to ESKD (1 received APD, while 1 received AHD) and eventually required a chronic dialysis program and possible future renal transplantation. Only 1 (1.6%) with CAKUT was switched to chronic hemodialysis as indicated in Tables 2 and 3. Out of the total of 11 patients who required chronic dialysis program or renal transplantation for ESKD, only 1 (9.1%) accessed chronic hemodialysis, giving a dialysis acceptance rate of 9.1%. The overall outcome showed the following: 9 (14.1%) improved and were discharged to be followed up at the clinic; 1 (1.6%) improved and was discharged to be on chronic dialysis program and clinic follow-up while awaiting renal transplantation outside the country; 10 (15.6%) were unable to sustain chronic dialysis program or access renal transplantation because of financial constraints and were lost to follow-up; 33 patients (51.5%) could not access RRT because of financial limitations and were discharged against medical advice (DAMA); 6 (9.4%) died on admission while sourcing for funds to access the therapy; 5 (7.8%) died while on RRT as shown in Table 4. Of the 5 (7.8%) patients who died on treatment, 4 (6.2%) patients had AKI 2^0^ to severe sepsis and 1 (1.6%) patient had AKI 2^0^ to postcardiac surgery complications. They all died from the complications of the primary disease within 24 hours of commencing RRT treatment as well as from late access to therapy. There were no deaths attributable to the complications of RRT during the period under review.

## 4. Discussion

RRT, either on acute or chronic basis, remains inaccessible and unaffordable in many of the developing countries of the world and has contributed to the high morbidity and mortality reported in studies from these regions [9–11, 15–17]. In this study, the calculated RRT requirement rate was 33% children, population with renal disease, with access and acceptance rates of 39.1% and 9.1%, respectively. The low access and acceptance rates were basically due to the financial constraints of the patients' parents/caregivers, majority of whom belonged to the low SECS with an average wage of less than $150 per month (Nigeria's National Minimum Wage [18]). Moreover, medical expenditure had to be paid out of pocket, without any health insurance scheme resulting in household financial catastrophe [19]. This exorbitant health care expenditure for RRT resulted in the overall outcome of low rate of treatment, low access and acceptance rates, loss to follow-up, high rate of discharge against medical advice in the current study, and death due to late access to RRT.

Furthermore, the dialysis access rate in this study is still low but slightly higher than the figures noted in previous studies conducted in this country where rates of 10% and 22.2% were documented [16, 20]. The marginal increase in the dialysis access rate in the index study could be due to the availability of free dialysate fluids and consumables donated by philanthropic groups and support by international organizations (such as International Pediatric Nephrology Association (IPNA) and International Society of Nephrology (ISN)). The dialysis acceptance rate in this study is low and the chance of accessing renal transplantation either locally or abroad is extremely difficult. This finding is similar to what is obtainable in most of the developing countries of the world especially in many of the sub-Saharan African countries [9–11, 21].

AKI with variable combinations of factors was the prominent indication for RRT in this study which is similar to the finding of other studies from the region as acute gastroenteritis with severe dehydration complicated by hypovolemic shock as well as severe infections continued to be the leading causes with resultant high morbidity and mortality [16, 17, 20, 22].

Most of the patients that accessed RRT in this index study received peritoneal dialysis (PD) as against hemodialysis (HD). PD is a less costly modality of RRT that can be adopted globally by low- and middle-income countries and can reduce the cost of RRT if PD consumables can be manufactured regionally or locally. Moreover there is presently a paradigm shift favoring the choice of PD globally due to its better acceptability by patients, better residual renal function, and its choice as a preferred mode of RRT in children. However, the cost of transportation of PD fluids and consumables has made PD more expensive than HD in some parts of the region [23]. Currently, the unit where this study was carried out is prospecting a collaboration with an indigenous pharmaceutical company based in the southeastern part of Nigeria for the possibility of producing the PD fluids and the consumables. However, there is a need for an international collaboration to consolidate and sustain this arrangement.

In conclusion, with high cost of renal health care and the prevailing depressed economy, the management of a failed kidney which requires either acute or chronic RRT in children in the developing countries of the world is froth with diverse challenges which contribute to the overall poor outcome of renal disease as well as the high general mortality and morbidity in the region. It is pertinent to consider renal disease in children as a global public health priority with emphasis on and the need for formulation of policies by national and international organizations and the government in order to alleviate the inaccessibility and unsustainability of RRT in children in such resource-limited setting. The global effort of preventing chronic kidney disease especially in low-resource countries of sub-Saharan African cannot be overemphasized.

## Figures and Tables

**Table 1:** The RRT required rate amongst the patients on admission.

Gender	Number of patients on admission	Number of patients on admission with renal disease	Number of patients who required RRT amongst those with renal disease
	*n* (%)	*n* (%)	*n* (%)
Male *n* (%)	2,086 (59.3%)	132 (3.7%)	35 (18.0%)
Female *n* (%)	1,434 (40.7%)	62 (1.8%)	29 (15.0%)

Total *N* (%)	3,520 (100%)	194 (5.5%)	64 (33.0%)

**Table 2:** Etiological diagnosis in patients who required RRT.

Etiological diagnosis	Number of patients who required RRT	Number of patients who accessed acute RRT	Number of patients who required further chronic RRT	Number of patients who accessed chronic RRT
	*n* (%)	*n* (%)	*n* (%)	*n* (%)
AKI	41 (64.1%)	18 (28.1%)	4 (6.3%)	1 (1.6%)
End stage renal disease (ESRD) 2^0^ chronic glomerulonephritide	12 (18.7%)	5 (7.8%)	5 (7.8%)	0 (0%)
Chronic kidney disease/ESRD 2^0^ unidentifiable	8 (12.5%)	2 (3.1%)	2 (3.1%)	0 (0%)
ESRD 2^0^ HIV nephropathy	3 (4.7%)	0 (0%)	0 (0%)	0 (0%)

Total	64 (100%)	25 (39.0%)	11 (17.2%)	1 (1.6%)

**Table 3:** Primary etiological diagnosis in AKI patients who required RRT.

Etiological diagnosis	Number of patients who required RRT	Number of patients who accessed acute RRT	Number of patients who required further chronic RRT	Number of patients who accessed chronic RRT
	*n* (%)	*n* (%)	*n* (%)	*n* (%)
Acute kidney injury (AKI) 2^0^ severe dehydration/acute gastroenteritis	16 (39.0%)	4 (9.8%)	0 (0%)	0 (0%)
AKI 2^0^ severe sepsis	10 (24.4%)	6 (14.6%)	0 (0%)	0 (0%)
AKI 2^0^ acute tubular necrosis (massive hemoglobinuria)	3 (7.3%)	2 (4.9%)	0 (0%)	0 (0%)
AKI 2^0^ tumor lysis syndrome	2 (4.9%)	0 (0%)	0 (0%)	0 (0%)
AKI 2^0^ toxic nephropathy	5 (12.2%)	2 (4.9%)	2 (4.9%)	0 (0%)
AKI 2^0^ acquired obstructive uropathy (nephrolithiasis)	1 (2.4%)	1 (2.4%)	0 (0%)	0 (0%)
AKI 2^0^ acute tubular necrosis (after cardiac surgery)	1 (2.4%)	1 (2.4%)	0 (0%)	0 (0%)
AKI 2^0^ congenital anomalies of the kidney and urinary tract- (CAKUT-) posterior urethral valves	3 (7.3%)	2 (4.9%)	2 (4.9%)	1 (2.4%)

Total	41 (100%)	18 (43.9%)	4 (9.8%)	1 (2.4%)

**Table 4:** The overall outcome of patients who required RRT.

Outcome of patients	Frequency	Percentage
	*n*	(%)
Improved and discharged on follow-up (RRT accessed)	9	14.1
Improved and discharged on chronic dialysis program (awaiting renal transplantation abroad)	1	1.6
Loss to follow-up (RRT accessed but unable to sustain chronic therapy/renal transplantation abroad unaccessed)	10	15.6
Discharged against medical advice (RRT unaccessed)	33	51.5
Demised while in the hospital (RRT accessed)	5	7.8
Demised on admission (RRT unaccessed)	6	9.4

Total	64	100
